# Supplementary material for: Improved estimation of time-varying reproduction numbers at low case incidence and between epidemic waves
Source: PLoS Comput Biol. 2021 Sep 7;17(9):e1009347. doi: 10.1371/journal.pcbi.1009347 (PMC8448340; doi:10.1371/journal.pcbi.1009347)
Supplement: S1 Appendix — This includes analyses of computational complexity, further examples showing performance and calculation of coverage statistics (additionally supporting Figs 2–5 of the main text). Results are embedded within Figs A-D of the S1 Appendix. Fig A: EpiFilter mechanics. We examine the computational complexity (as a function of grid size m on the examples in Figs 6 and 7) and accuracy (in terms of m and state noise η) of EpiFilter. Fig B: Validation against APEestim. We compare estimates from EpiFilter with optimised ones from APEestim [13] over various general epidemic scenarios (which may have large or small incidence). Fig C: Coverage statistics of waning epidemics We provide the probability that the true values of instantaneous reproduction numbers and incidence from scenarios in Fig 3 are within EpiFilter’s 95% credible intervals. Fig D: Coverage statistics of resurging epidemics. We provide the probability that the true values of instantaneous reproduction numbers and incidence from scenarios in Fig 5 are within EpiFilter’s 95% credible intervals. (PDF) [file pcbi.1009347.s001.pdf]

S1 Appendix: Validating the mechanics and performance of EpiFilter

Kris V Parag*, [1]

[1]MRC Centre for Global Infectious Disease Analysis, Imperial College London, London, UK
*Email: k.parag@imperial.ac.uk

Having constructed EpiFilter (see Methods) we now investigate its mechanics and performance. First we show how the choice of grid resolution, m, affects the accuracy and computational complexity of our smoothed instantaneous reproduction number estimates, $\hat{R}_s$. Our recursive implementation is also known as a grid smoother, since its estimates are optimal (in a MSE sense) but conditioned on the grid $\mathcal{R}$ [1], [2]. In the left panels of Fig A we reconsider the empirical H1N1 and COVID-19 examples, from the main text, at various m. We find that EpiFilter has linear complexity in m but that $\hat{R}_s$ (averaged over 50 replicate runs) converges quickly, both in mean and variance (latter not shown). Execution times, which include both R_s estimation and incidence (I_s) prediction, are only a few minutes, generally. While the R_s smoother is always fast, the I_s prediction block may be slower for very large epidemics (e.g., 1000s of cases per day), resulting in ~ 10 minute run times.

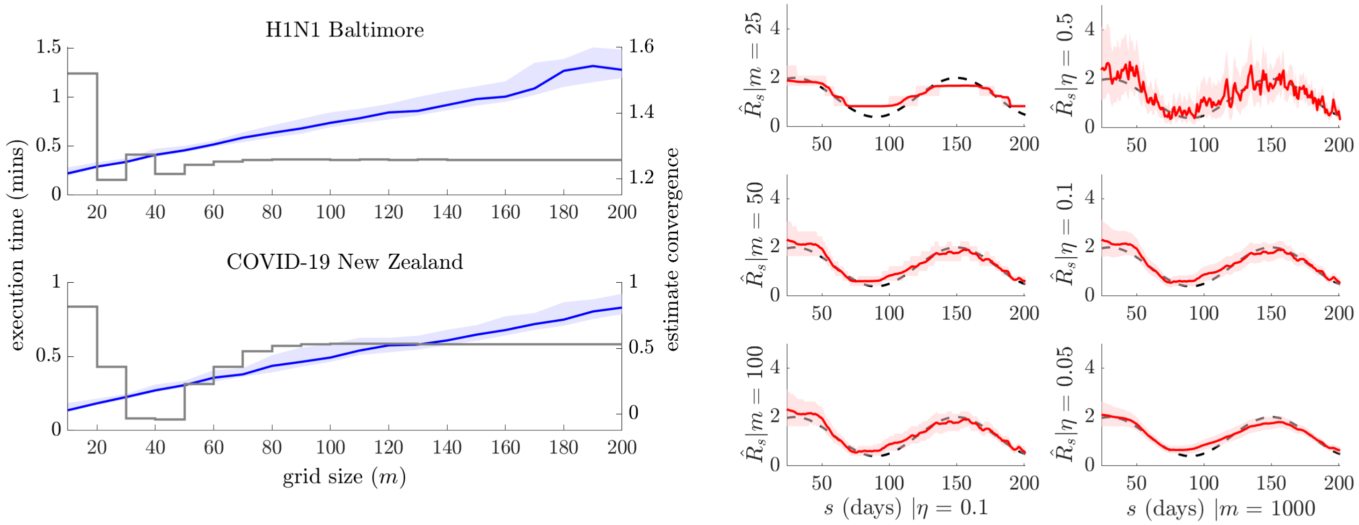

Fig. A: **EpiFilter mechanics.** We examine the computational complexity and accuracy of EpiFilter. Left panels indicate how increasing grid resolution, m, leads to a linear increase in execution time (blue with 95% credible intervals) for the empirical case studies of the main text. These times are averaged over 50 replicate runs (using the R implementation) and include the latency in computing both reproduction number, R_s, estimates and incidence, I_s, predictions. The mean estimate of R_s, averaged over time and the replicates, is in grey, and converges quickly. The variance of these estimates, while not shown, converges equally fast. Right panels provide an illustrative example of how estimates $\hat{R}_s$ (red with 95% credible intervals) change with m and choices of process noise parameter, η. Even at coarse grid sizes (small m) the estimates are sensible. Reducing η sharpens confidence intervals but can potentially underfit. Increasing η adds state noise. We find that $\eta = 0.1$ serves as a good general heuristic. All simulations are at $R_{\text{min}} = 0.01$ and $R_{\text{max}} = 10$ and the true R_s (a seasonal epidemic) is in black.

As a rule-of-thumb setting $m \geq 10^3$ safely guarantees convergence, and still executes in a few minutes. Note that estimates at small m are still valid. They represent coarser assumptions on reproduction numbers and provide minimised MSE estimates under those assumptions. We illustrate a coarse estimate and how it converges for a simulated example in the right panels of Fig A. There, we also test the behaviour under different choices of the process or state noise parameter η. This parameter is vaguely similar to the window size parameter, k, in EpiEstim

approaches, but is easier to select. It controls the autocorrelation among successive R_s and allows some modelling of heterogeneity (hence the term state noise). Fig A shows how too small η might lead to underfitting, while overly large η (where we would not consider $\eta \geq 1$ given Eq. (4b) of the main text) could promote overfitting.

However, while the optimal window size in EpiEstim varies with the form of the true R_s (e.g., large k is needed for stable R_s time-series and small k for strongly fluctuating ones [3]), we find that a fixed $\eta = 0.1$ works well across diverse epidemic scenarios and enables automatic detection of transmission change-points. This is an advantage over approaches requiring explicit change-point modelling [4]. We examine this further in Fig B. There we compare R_s estimates from APEestim (with optimal window k) [3] to EpiFilter ($\eta = 0.1$, $m = 2000$) for simulated epidemic scenarios. We generate epidemics via the renewal model (Eq. (1) of the main text) using the Ebola virus serial interval distribution from [5] and ensure the prior distributions of both methods have the same support. The epidemics simulated span the range of possibilities (e.g., they may have large or small incidence).

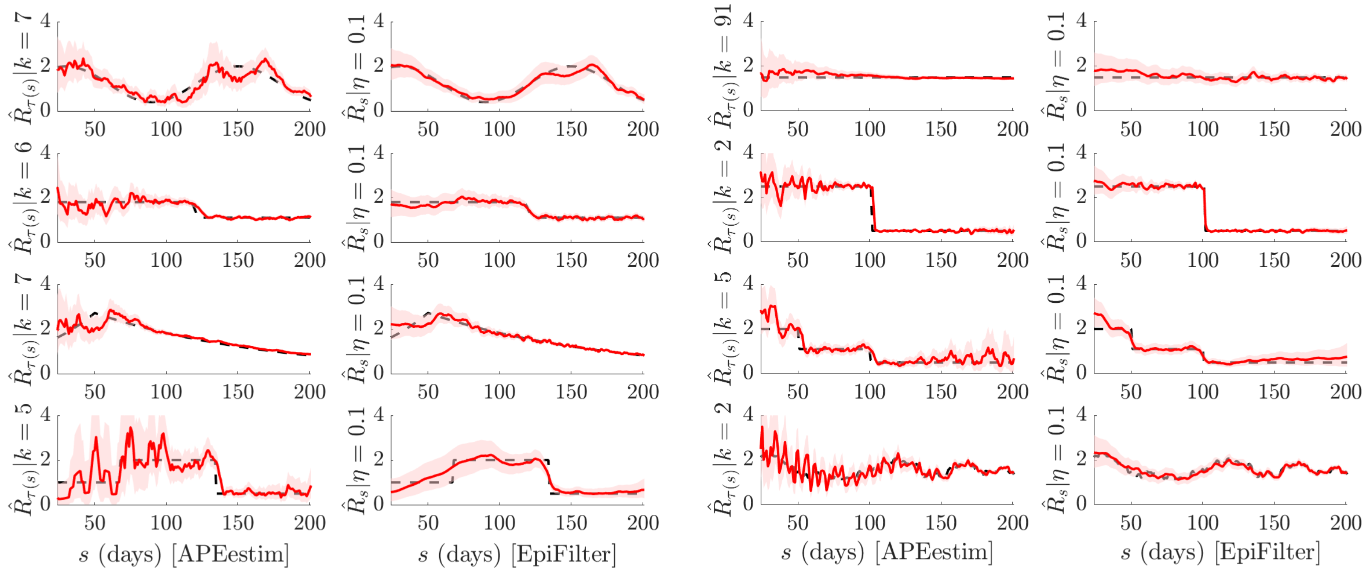

Fig. B: **Validation against APEestim.** We compare estimates from EpiFilter (right panels) with optimised ones from APEestim (left panels [3]) over various general epidemic scenarios (which may have large or small incidence). These are simulated using Eq. (1) of the main text and not restricted to epidemics that may face elimination or resurgence as in Fig 2 and Fig 4 of the main text. We use $m = 2000$, $R_{\text{min}} = 0.01$ and $R_{\text{max}} = 10$ for EpiFilter. To be fair we use a $\text{Gam}(1, 2)$ prior distribution with APEestim, which sums to 1 by R_{max}. Here k is the optimal window choice in APEestim (under predictive error) and $\eta = 0.1$ is fixed for EpiFilter inferences. True R_s is in black and conditional mean estimates with 95% credible intervals are in red. EpiFilter is more stable, smoother and minimises estimate uncertainty in all scenarios. While the optimal k changes considerably among epidemics, $\eta = 0.1$ is able to automatically detect salient changes in transmission for all examples considered.

We find EpiFilter outperforms APEestim, despite our using a single η value, with improvements especially stark for the simulations with subcritical transmission (as detailed in main text). This single η contrasts the discordant optimal k values and allows EpiFilter to recover the salient dynamics for epidemics with stable trajectories, seasonal variations and rapidly or gradually fluctuating transmission. In none of these scenarios does the true R_s have either the state model of Eq. (4b) of the main text or the sliding-window relationship in EpiEstim. Hence, these simulations also indicate some robustness of the different inherent state assumptions in both approaches to model mismatch. We use single runs to showcase how a practical epidemic would be processed in real-time. Neither method depends on random algorithms (e.g., MCMC) so their estimates will remain constant under the same parameters.

Last, we complete our batch analysis of EpiFilter performance by examining coverage statistics. In Fig 3 and

Fig 5 of the main text several waning and resurging epidemic examples were explored. There we found that EpiFilter markedly reduces the MSE when inferring instantaneous reproduction numbers, R_s, relative to EpiEstim and APEestim. APEestim, expectedly, had the smallest PMSE when predicting incidence, I_s, but EpiFilter was only marginally worse. In Fig C and Fig D we plot the coverage (over the 200 runs of every scenario in Fig 3 and Fig 5) against time i.e., the probability that the R_s estimate or I_s prediction equal tailed credible intervals of a given method contain the true R_s and I_s values from the simulations. We find that EpiFilter generally has the most consistent coverage across scenarios for both R_s and I_s, confirming its performance.

The one blip in EpiFilter's coverage (at the change-point in 4A) follows from it not reverting to its prior whereas the other methods do. While EpiFilter does sometimes have higher than 95% coverage, this likely follows from the additional overdispersion it introduces (relative to EpiEstim, APEestim and the ground truth). When combined with Figs 2-5 of the main text, the overall picture shows that EpiFilter improves reproduction number estimates. The EpiFilter package is available (R and Matlab implementations) at https://github.com/kpzoo/EpiFilter.

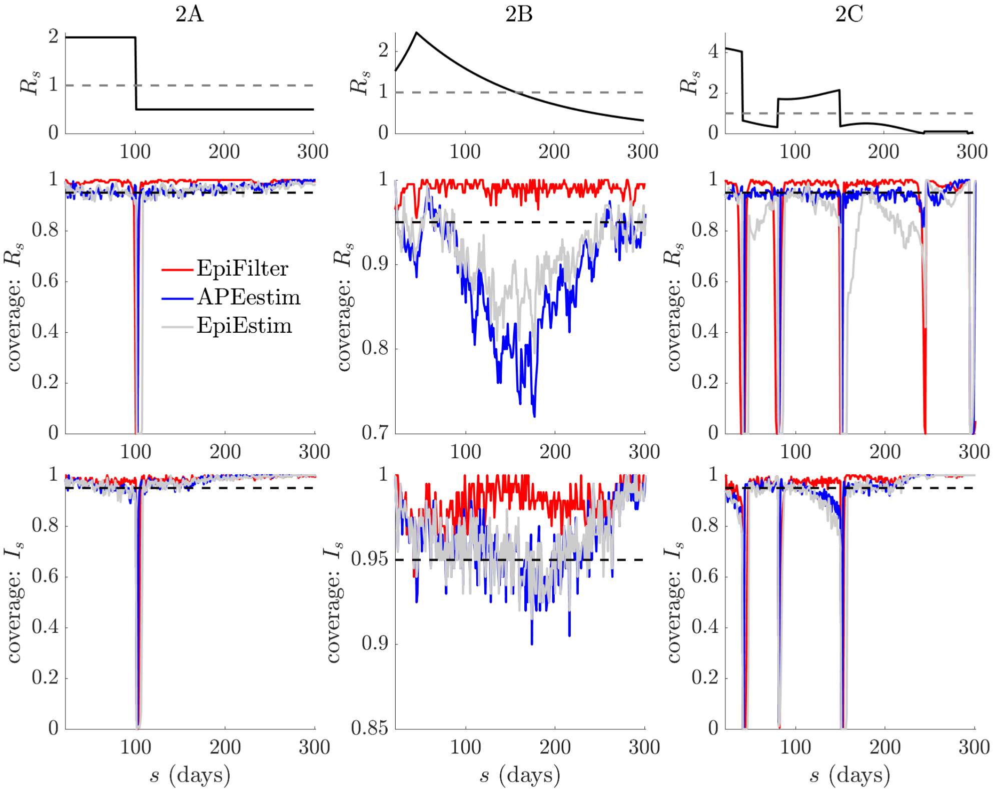

Fig. C: **Coverage statistics of waning epidemics** We provide the probability that the true values of instantaneous reproduction numbers, R_s, and incidence, I_s, from each scenario in Fig 3 of the main text (over 200 runs) are contained within the equal tailed 95% estimate and prediction credible intervals generated by EpiFilter ($\eta = 0.1$), APEestim ($k = k^*$) and EpiEstim (weekly, $k = 7$ windows). EpiFilter provides the most consistent coverage.

References

1. Sarrka S. Bayesian Filtering and Smoothing. Cambridge, UK: Cambridge University Press; 2013.
2. Parag K, Pybus O. Exact Bayesian Inference for Phylogenetic Birth-death Models. Bioinformatics. 2018;34(21):3638–45.
3. Parag K, Donnelly C. Using information theory to optimise epidemic models for real-time prediction and estimation. PLOS Comput Biol. 2020;16(7):e1007990.

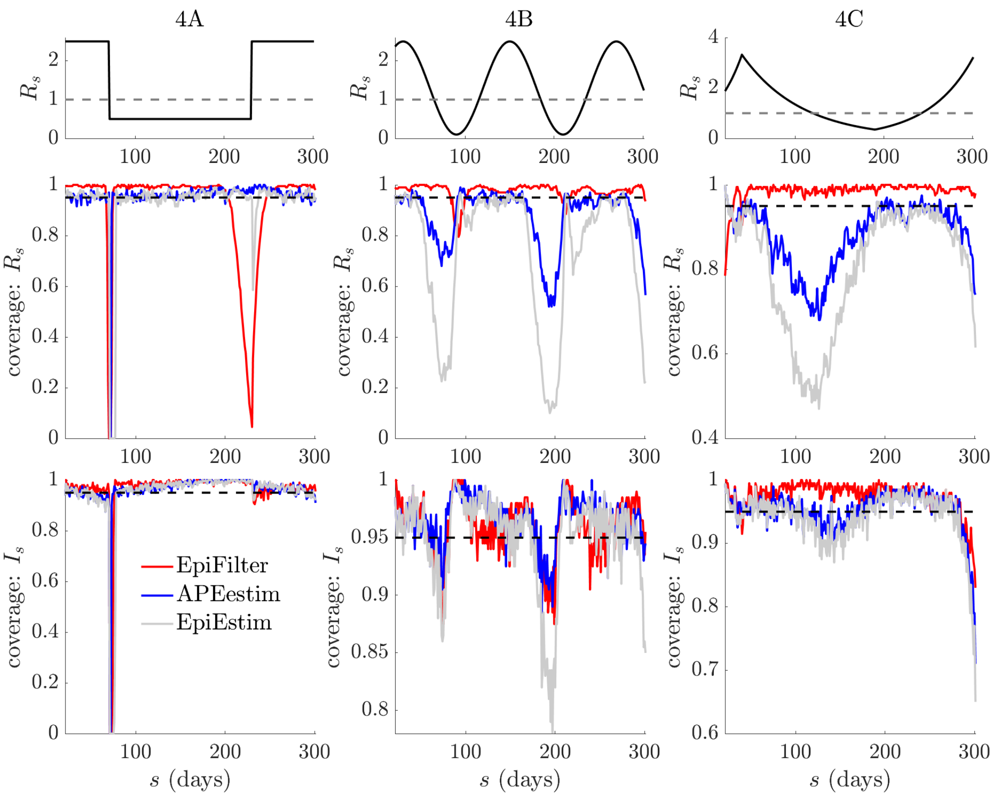

Fig. D: **Coverage statistics of resurging epidemics.** We provide the probability that the true values of instantaneous reproduction numbers, R_s, and incidence, I_s, from each scenario in Fig 5 of the main text (over 200 runs) are contained within the equal tailed 95% estimate and prediction credible intervals generated by EpiFilter ($\eta = 0.1$), APEestim ($k = k^*$) and EpiEstim (weekly, $k = 7$ windows). EpiFilter provides the most consistent coverage.

4. Flaxman S, Mishra S, Gandy A, Unwin H, Coupland H, Mellan T, et al. Estimating the effects of non-pharmaceutical interventions on COVID-19 in Europe. Nature. 2020;584:257–261.
5. Van Kerkhove M, Bento A, Mills H, Ferguson N, Donnelly C. A review of epidemiological parameters from Ebola outbreaks to inform early public health decision-making. Sci Data. 2015;2:150019.
